# Spider Web Architecture and Rainfall Damage: Observational and Manipulative Studies Along a Precipitation Gradient on the Tropical Andes

**DOI:** 10.1002/ece3.73432

**Published:** 2026-04-07

**Authors:** Yu‐Heng Lin, Antonio Domingos Brescovit, Leticia Avilés

**Affiliations:** ^1^ Department of Zoology and Biodiversity Research Centre University of British Columbia Vancouver British Columbia Canada; ^2^ Laboratório de Coleções Zoológicas Instituto Butantan São Paulo SP Brazil

**Keywords:** microhabitat use, rainfall intensity, spider webs, tropical Andes, web architecture, web damage

## Abstract

Rainfall is a key abiotic factor influencing species' overall fitness and their ecological interactions. Although rainfall's effects are expected to be generally positive, high‐intensity rainfall can damage biological structures and thereby reduce fitness. As silk structures should be particularly vulnerable to heavy rainfall, web‐building spiders can serve as ideal systems for examining the impacts of rainfall on organisms and their extended phenotypes. Here, we investigated how spider webs with different geometries—two‐dimensional (2D) orbs and three‐dimensional (3D) tangles and sheet‐and‐tangles—are affected by varying levels of rainfall intensity given both their physical structure and microhabitat use. We studied the impact of rainfall on spider webs along an elevational gradient on the eastern slopes of the Tropical Andes in Ecuador where rain intensity ranges from strong (> 4 mm/h) to mild (< 2 mm/h) from the lowland rainforests to higher‐elevation cloud forests. We found that web damage significantly increased with rainfall intensity, but the amount of damage webs suffered differed for different web types. Orb webs, which are built in open microhabitats, had the highest probability of damage. Sheet‐and‐tangle webs, on the other hand, suffered the greatest material loss, likely due to their high silk content, despite being located in more protected microhabitats. A manipulative experiment showed that webs artificially protected from the rain suffered significantly less damage than those left unprotected, demonstrating the role of immediate cover in mitigating the impact of rainfall as a function of microhabitat use. Our findings demonstrate that spiders with different web architectures are differentially affected by heavy rainfall, thus highlighting the need to consider rainfall intensity as a factor determining the composition of web‐building spider communities across precipitation gradients.

## Graphical abstract

Rainfall intensity can impose substantial abiotic stress on organisms and their extended phenotypes. Using field observations and a manipulative experiment along an elevational gradient in the tropical Andes, we examined how rainfall intensity damages spider webs of different types. Web damage increased with rainfall intensity but differed among web types based on their architecture and microhabitat use: orb webs were the most frequently damaged due to their open exposure, but sheet‐and‐tangle webs suffered the greatest material loss due to their high silk content. These findings underscore the importance of rainfall intensity, not just rain volume, in shaping the composition of web‐building spider communities.

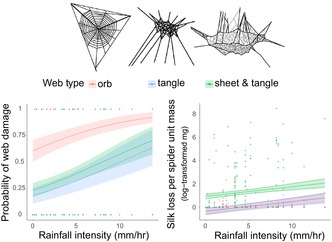

## 1 Introduction

Rainfall is expected to play a key role in shaping the composition of animal communities across ecosystems (Gutiérrez‐Hernández and García 2021; Robertson and Avilés 2019; Srivastava et al. 2020). Rainfall may influence ecosystems in positive and negative ways: on the one hand, it increases primary production and thus species abundances (Xie et al. 2023); on the other, it may reduce species diversity and evenness (Bento et al. 2017). In arid and semiarid ecosystems, for instance, rainfall has been shown to play a role in shaping arthropod multi‐trophic interactions (Fischer et al. 2022) and to interact with grazing intensity in shaping above ground biomass and plant community composition (Wan et al. 2015). Rainfall patterns may also affect the morphology and behavior of organisms, as well as their overall fitness, as species must adapt to fluctuating and sometimes extreme conditions. Despite its significance, research on the impacts of rainfall intensity remains limited (Pires et al. 2016). In an era of anthropogenic climate change, studying these dynamics, however, becomes particularly urgent given the increasing frequency of extreme weather events (Mora et al. 2017; Prein et al. 2017) and unpredictable rainfall patterns (IPCC 2021).

**FIGURE 1:**
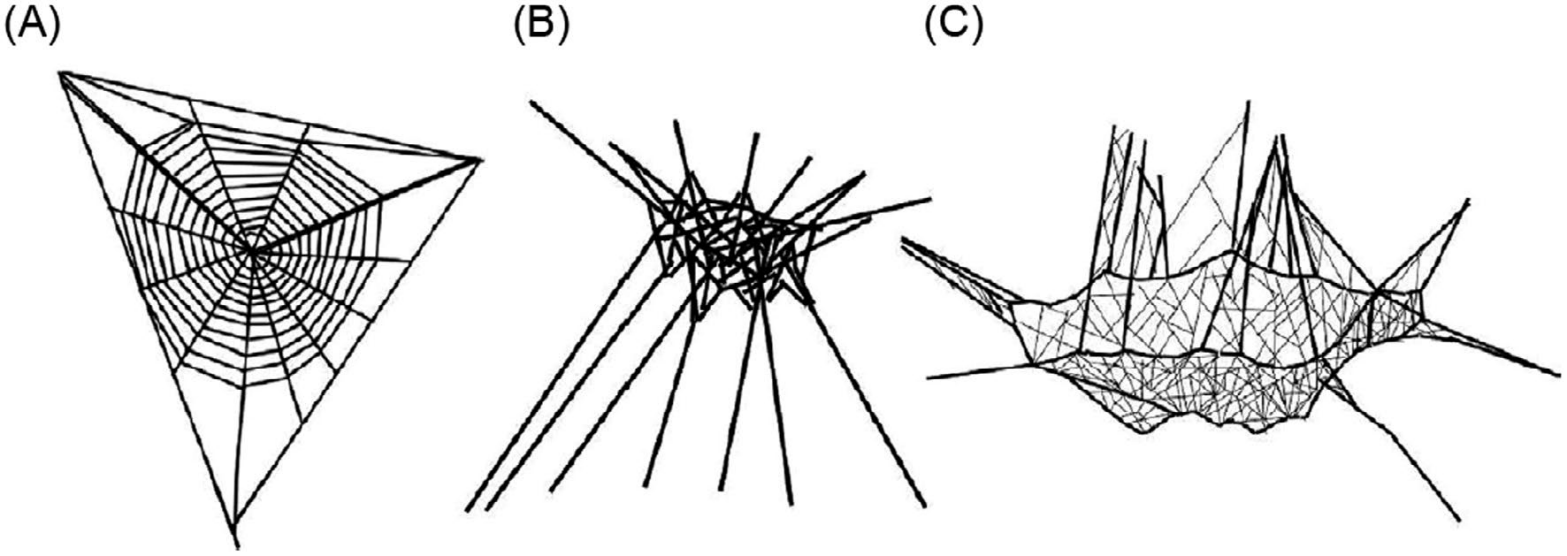
Examples of (A) an orb web, (B) tangle web, and (C) sheet‐and‐tangle web. Drawings from Straus et al. (2022).

The torrential rains of the lowland tropical rainforest are of particular interest as they may directly or indirectly (e.g., through falling branches or trees) damage plant and animal structures. Fragile structures, such as the webs spiders build, may be particularly vulnerable. Spider webs are extended phenotypes that, while exhibiting taxon‐specific features, must adapt dynamically to capture different types of prey and to respond to changing environmental conditions (Blamires 2010). Web‐building spiders thus serve as an appropriate system to test how heavy rainfall may impact organismal fitness. The architecture of spider webs, which influences their functionality and resilience, can be categorized into three broad types: two‐dimensional (2D) orbs and three‐dimensional (3D) tangles and sheet‐and‐tangles (Janetos 1982; Rypstra 1982; Straus et al. 2022; Figure 1). 3D webs serve multiple purposes, functioning both as tools for prey capture and as structures that house the spiders and provide protection from predators (Blackledge et al. 2003). Any additional benefits notwithstanding, some types of 3D webs represent a massive material investment for the spiders that build them. Straus et al. (2022) showed that 3D sheet‐and‐tangle webs, in particular, contain two orders of magnitude more silk per unit spider mass than 2D orbs or simple 3D tangles. As a result, 3D weavers rarely relocate their webs (Janetos 1982; Tanaka 1989), whereas 2D orb weavers relocate frequently and may respond to web damage by performing small repairs (Tew et al. 2015) or replacing the entire web (Blackledge 2011; Blackledge et al. 2011) (Table S1). The substantial silk content of sheet‐and‐tangle webs makes them particularly vulnerable to the effects of heavy rainfall, as damage could require costly repairs.

Despite their potentially greater costs of repair or replacement, Robertson and Avilés (2019) found that the frequency of 3D webs increased from dry coastal to wet rainforest areas in western Ecuador. This pattern was at odds with the prediction that 3D webs would be more greatly affected by rainfall damage but consistent with the protective role of 3D webs against predators, as ants—the main predators of small arthropods in the tropics (Camacho and Avilés 2019; Floren et al. 2002; Novotny et al. 1999)—were more active and abundant in wetter areas (Robertson and Avilés 2019). As a potential solution to the apparent dilemma of costly 3D webs being more common where exposed to stronger rains, Robertson and Avilés (2019) found that as leaf size increased along with rainfall, 3D weavers tended to be covered by larger leaves in wetter areas. Spiders may thus maximize their fitness by optimizing their microhabitat use (Goldsbrough et al. 2004; Lubin et al. 1993), with 3D weavers positioning their webs under the shelter of leaves (Haberkern et al. 2020). 3D webs in wet tropical habitats may thus provide predator protection while minimizing the energetic costs associated with web damage through microhabitat choice (Haberkern et al. 2020; Robertson and Avilés 2019). The extent of damage from rainfall webs suffer as a function of their architecture, silk content, and microhabitat use, however, remains to be determined.

We addressed the above question along a gradient of precipitation intensity on the eastern slopes of the Tropical Andes in Ecuador where rainfall ranges from intense (> 4 mm/h) in the lowland tropical rainforest to mild (< 2 mm/h) at higher elevations (Hoffman and Avilés 2017; this study). Spiders with costly 3D webs in these habitats may avoid significant costs by using naturally protected microhabitats, such as under leaves or against tree trunks (Haberkern et al. 2020). Nonetheless, given the large amount of silk 3D sheet‐and‐tangle webs contain (Straus et al. 2022), spiders with this web architecture may suffer significant costs as a result of web damage. To test these hypotheses, we conducted observational and manipulative studies along the eastern slope of the Tropical Andes in Ecuador, examining how rainfall intensity impacts webs of different architectures in terms of direct material damage as a function of their associated microhabitat use. We combined the observational study with a manipulative experiment to test the role of immediate cover in providing protection against rainfall damage. We conducted the manipulative experiment in the lowland tropical rainforest using artificial protection to create additional immediate cover. Our findings illustrate how the behavior of organisms and the structure of their extended phenotypes may be impacted by rainfall of different intensities and provide insights into how microhabitat selection may interact with abiotic factors to shape the distribution of spider web architectures along rainfall gradients.

## 2 Materials and Methods

### 2.1 Study Site

**FIGURE 2:**
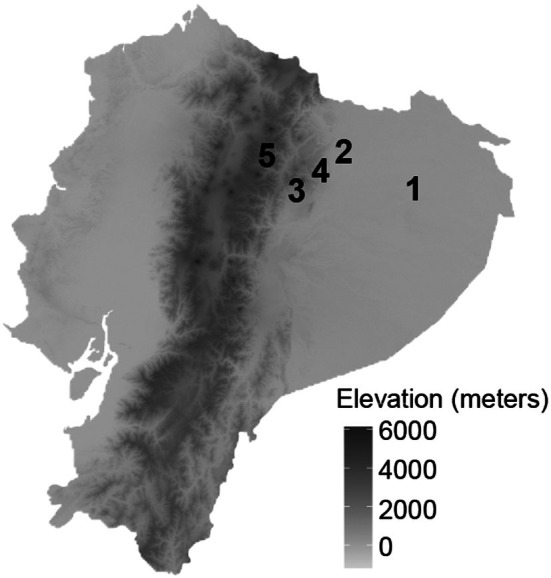
Study sites along the elevational gradient in eastern Ecuador. Map showing the locations of the five study sites across the elevational gradient, including the (1) Yasuní National Park (3.31 mm/h), (2) Bigal River Biological Reserve (4.13 mm/h), (3) Narupa Reserve of the Jocotoco Foundation (2.12 mm/h), (4) Yanayacu Biological Research Station (2.87 mm/h), and (5) Cayambe‐Coca Papallacta (1.27 mm/h). Coordinates included in Table S2.

We conducted the study during June–July 2022, which corresponds to one of the wetter periods of the annual rainfall cycle in this region (Hoffman and Avilés 2017). The study was carried out at five sites on the eastern slopes of the Ecuadorian Andes (Figure 2), where rainfall intensity ranged from 1.27 mm/h at the highest elevation (Papallacta, at 3440 m) to 4.13 mm/h at the second lowest elevation (the Bigal River Biological Reserve, at 960 m elevation) (Table S2). For the manipulative experiment, we conducted the study at the lowest elevation site, Yasuní National Park, which had the second highest rain intensity (3.31 mm/h). We selected this site because its relatively high rainfall intensity provided suitable conditions for testing the effects of immediate cover under strong rainfall.

### 2.2 Data Collection

Across at least two distinct areas within each study site, we sampled at least 30 spiders (up to 55) and recorded the effects of at least three rainfall events per web. In total, we examined 207 webs in the observational study and 86 webs in the manipulative experiment, representing 20 genera across 10 spider families (Table S1). We classified webs based on their geometry (Janetos 1982; Rypstra 1982; Straus et al. 2022) rather than their taxonomy, as some genera in orb‐weaving families, such as *Kapogea*, combine horizontal barrier webs with vertical knock‐down capture lines and were thus classified as 3D webs. Our field sites also contained web‐building spider genera that belong to wandering spider families, such as *Aglaoctenus* (Lycosidae) or *Architis* (Pisauridae), both of which build sheet‐and‐tangle webs with high silk content (Table S1). For each web, we recorded the spider's life stage (juvenile or adult) and initially estimated body length in mm (from the front of the chelicera to the tip of the abdomen) in the field. When specimens could be collected, these estimates were later updated with measurements to the nearest 0.1 mm taken under a microscope in the laboratory. We also recorded the web type, density of the prey capture threads (number of threads/cm), web dimensions (longest axis and width, for orb webs; length of the longest horizontal axis, width and height, for tangle; length and width of the sheet and the height of the prey capture threads above it, for sheet‐and‐tangle webs, in mm); documented the web's microhabitat use, defined as the type of immediate cover (IMC) within 25 cm above the web (no cover, under a single leaf, under multiple leaves, or against a tree trunk; following Haberkern et al. 2020). The 25 cm threshold corresponds to the spatial scale at which overhead vegetation can directly intercept rainfall and influence web exposure. We assessed the condition of each web at the time of data collection (e.g., poor, fair, or good), and estimated web damage immediately following a rainfall event as the percentage web lost during the event. Because web architectures differ fundamentally in geometry and structure, a single standardized surface‐area metric could not be applied across all web types. Instead, we used architecture‐specific measures that quantify proportional structural loss relative to the original web. For orb webs, we divided the damaged surface area by the total web area prior to the event. For tangle webs, where damage cannot be assessed by surface area, we calculated the percentage loss of silk density (silk/cm). Silk density was defined as the number of vertical silk threads on the horizontal medial axis of the tangle web. For sheet‐and‐tangle webs, we divided the damaged sheet area by the total sheet area prior to the rainfall event. Damage was assessed using architecture‐specific measures due to structural differences among web types, each measure reflects proportional loss relative to the original web. To estimate silk lost per unit spider mass, we inferred a spider's dry mass (M, mg) from its length (L, mm) using the equation from Straus and Avilés (2018):

$$\log_{10}M=-1.918+3.368\;\log_{10}L,$$

obtained from a sample of tropical spiders ranging in body length from 4.0 to 11.0 mm. We then calculated mass‐specific silk content (S, silk mass per unit spider mass) following Straus et al. (2022), who estimated the dry weight of field‐collected tropical spiders and their webs. The relationship between silk content (S) and spider mass (M) was estimated as:

For sheet‐and‐tangle webs: $\log_{10}S=2.28-0.58\;\log_{10}M;$for orb and tangle webs: $\log_{10}S=-1.09-0.58\;\log_{10}M.$

Values were then back‐transformed to calculate the amount of silk lost per web and rainfall event per unit spider mass.

In the manipulative experiment, we examined 31 webs protected from rain by tarps and 55 unprotected as controls. In the manipulation group, we placed waterproof tarps measuring 1 m × 1 m at approximately 50 cm above the spider's web to simulate a protective cover. We paired each protected web with spiders in the same genus and similar body size, randomly assigned to either treatment. In both the observational study and the experiment, we recorded whether the orb webs were removed before and after rainfall as orb webs are typically recycled (Peakall 1971). When orb weavers abandoned their site, we sought additional spiders with similar characteristics to replace them to maintain our sample size.

We recorded rainfall data using a HOBO Rain Gauge Data Logger RG3 at all five study sites. We obtained hourly rain rate (mm per hour) throughout the observation periods (from approximately 4 h to overnight, depending on when rainfall events happened). Because structural damage to spider webs is likely driven by the mechanical impact of intense rainfall rather than cumulative precipitation, we utilized the rainfall event with the highest hourly rate at each site as a measure of rainfall intensity in our analyses. To examine rainfall differences across microhabitats within a study site, we placed manual rain gauges in microhabitat types as those used by the spiders: no cover, under a single leaf, under multiple leaves, and against a tree trunk. We calculated rainfall intensity under the different immediate cover types by dividing the total amount of rain collected in the gauge by the duration of the active rainfall events during the measurement period, as recorded by the HOBO rain gauge. These methods were consistent across both the observational study and the manipulative experiment.

### 2.3 Spider Identification

At the end of the observational study and manipulative experiment, we collected the spiders that remained on their webs and preserved them in 95% ethanol in compliance with our research permit (No. 00652; MAATE‐ARSFC‐2022‐2278). In the lab, we assigned every specimen a unique number based on their web ID. In addition to measuring the body length of collected specimens to the nearest 0.1 mm, we identified them to the lowest taxonomic level possible using the key to Amazonian spiders (Brescovit et al. 2002). A total of 101 specimens were identified to the genus level, while 76 specimens (predominantly juveniles) could only be identified to the family level. The specimens have been temporarily deposited in the arachnological collections of the Instituto Butantan, São Paulo (IBSP, curator: A.D. Brescovit).

#### 2.3.1 Statistical Analyses: Environmental Factors

We used a linear regression model to examine the relationship between rainfall intensity obtained with the HOBO rain gauge across the five study sites (average rain rate per hour after removing zeros). To analyze the rainfall data under different immediate cover types, we fitted a linear mixed‐effects model (LMM) with IMC type (no cover, under a single‐leaf, under multiple leaves, or against a tree trunk) as the explanatory variable, elevation (which correlates with rain intensity, Hoffman and Avilés 2017; this study) as a covariate, and location as a random factor. An interaction term between IMC type and elevation was not significant in an exploratory analysis and therefore excluded from the final model. We used post hoc Tukey tests to compare rainfall rates across different microhabitat types after controlling for elevation.

#### 2.3.2 Statistical Analyses: Web Damage and Web Types

To account for phylogenetic non‐independence among the taxa in our sample, we used a published spider phylogeny (Kulkarni et al. 2023) to reconstruct the relationships of the spider families included in our study (Figure S1). We identified three phylogenetically separate groups within the phylogeny of our study taxa, each containing representatives of all three web architectures (orb, tangle, and sheet‐and‐tangle). The criterion for phylogenetic separation was that the groups had no overlapping branches (Felsenstein 1985; Maddison 2000). We then carried out all analyses with the phylogenetically separate groups as a random factor in all mixed‐effects models for both the observational study and the manipulative experiment.

For the observational study, we used Fisher's exact tests to examine whether the association between web type and microhabitat type (no cover, under single leaf, under multiple leaves, against trunk) changed along the elevation gradient. We also compared the percentage of immediate cover and of canopy cover associated with each web type across the elevation gradient using linear regression models (with a transformed beta distribution for proportion data) followed by post hoc Tukey tests. We analyzed the probability of web damage and the proportion of web damage for the three web types as a function of rainfall intensity (highest rain rate during an observation period) across the five study sites. These analyses were performed using a generalized linear mixed‐effects model (GLMM) and a LMM with a transformed beta distribution, respectively. We used a linear mixed‐effects model to examine the relationship between log‐transformed silk loss per unit spider mass and rainfall intensity for each web type. We applied a LMM with a transformed beta distribution to analyze the proportion of web damage as a function of rainfall intensity for webs associated with the various microhabitat types. In all mixed‐effects models, spider body length was included as a covariate and spider web ID nested within study site was specified as a random factor to account for multiple observations per web. Post hoc Tukey tests were conducted to compare differences in web damage between immediate cover types and between web types.

In the manipulative experiment, we evaluated whether treatment type and rainfall intensity predicted the probability of web damage and the proportion of web damage for each web type. These analyses were conducted using a GLMM and a LMM with a transformed beta distribution, respectively. Spider body length was included as a covariate, and spider web ID was specified as a random factor in each model. Every continuous variable in each model was scaled to a mean of 0 and a standard deviation of 1 to avoid convergence issues. All models used in the analyses are included in Table S3 as a reference. All statistical models were implemented in the glmmTMB package (Brooks et al. 2017) using R version 4.5.1 (R Core Team 2024). All data and code required to reproduce the analyses are publicly available via Zenodo (see Data accessibility statement).

## 3 Results

**FIGURE 3:**
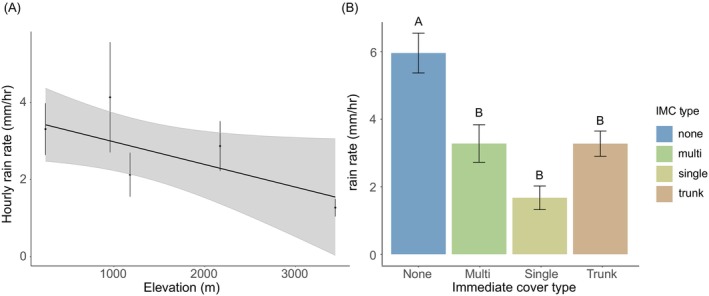
Hourly rainfall intensity along the elevational gradient and the rain rate under different microhabitat types. (A) Relationship between hourly rainfall intensity (mm/h) and elevation (m) showing a decrease in rainfall intensity with increasing elevation. The black line represents the fitted model, and the shaded area indicates the 95% confidence intervals. (B) Average rain rate (mm/h) under different immediate cover types (none, single, multi, and trunk) after controlling for elevation. Different letters indicate significant differences between categories using a post hoc Tukey test (Table S4).

Rainfall intensity across the five study sites showed a decreasing trend with elevation, when measured with the Hobo rain gauge (Figure 3A, *t*‐value = −1.9, *p* = 0.056). Across the elevation gradient, manual rain gauges showed that locations against tree trunks or under single or multiple leaves experienced significantly milder rain than locations without immediate cover (post hoc Tukey tests, Figure 3B, Table S4).

**FIGURE 4:**
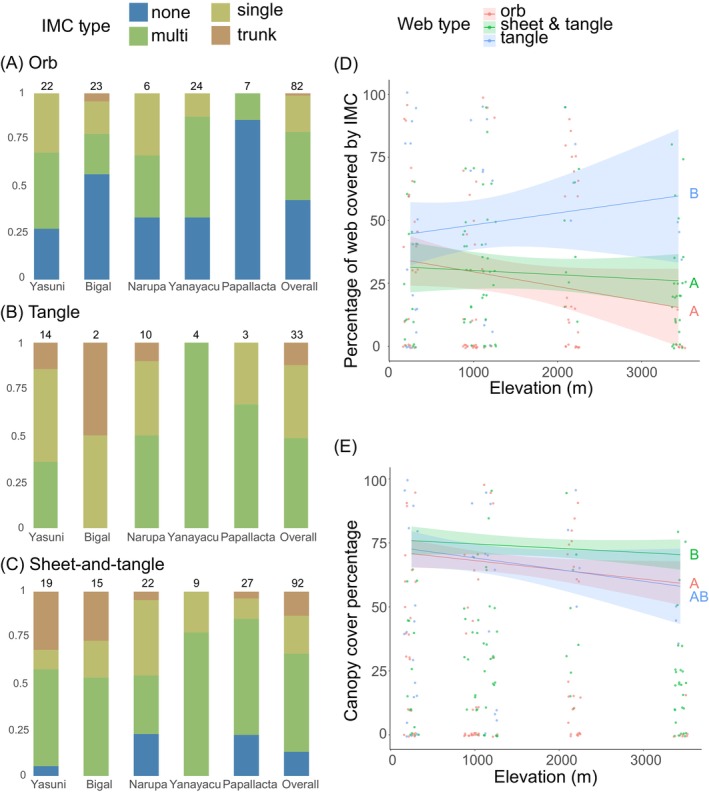
Microhabitat use and amount of immediate and canopy cover associated with different web types across the elevation gradient. Microhabitat use for spiders building (A) orb webs, (B) tangle webs, and (C) sheet‐and‐tangle webs across the five study sites in the observational study, with sites listed from lowest to highest elevation. Each bar represents the relative use of microhabitat types. The numbers above the bars represent the sample size (number of webs) in the observational study. IMC is the abbreviation of immediate cover. (D) Percent of a web's horizontal projection covered by immediate cover for each web type across the gradient. (E) Amount of canopy cover (percent cover) above different web types across the gradient. Different letters indicate significant differences between categories using a post hoc Tukey test. The shaded areas indicate 95% confidence intervals.

In the observational study, we included 207 webs at five sites along the elevational gradient. Across the gradient, orb weavers tended to build their webs in microhabitats without any immediate cover whereas tangle and sheet‐and‐tangle weavers tended to build their webs under leaves or against the tree trunks (Figure 4A–C). Analyzing each web type separately, we found that sheet‐and‐tangle weavers changed their microhabitat use patterns along the elevational gradient (Fisher's exact test, *p* = 0.009), with a greater representation of webs placed against tree trunks at the lowest elevations (Figure 4C). On the other hand, there were no significant differences in microhabitat use patterns for orb (Fisher's exact test, *p* = 0.089) and tangle weavers (Fisher's exact test, *p* = 0.38) across the elevation gradient. When testing the amount of immediate cover over different web types, we found that tangle webs were the most covered (post hoc Tukey tests, Figure 4D, Table S5) whereas sheet‐and‐tangle webs had the most canopy cover (post hoc Tukey tests, Figure 4E, Table S6).

**FIGURE 5:**
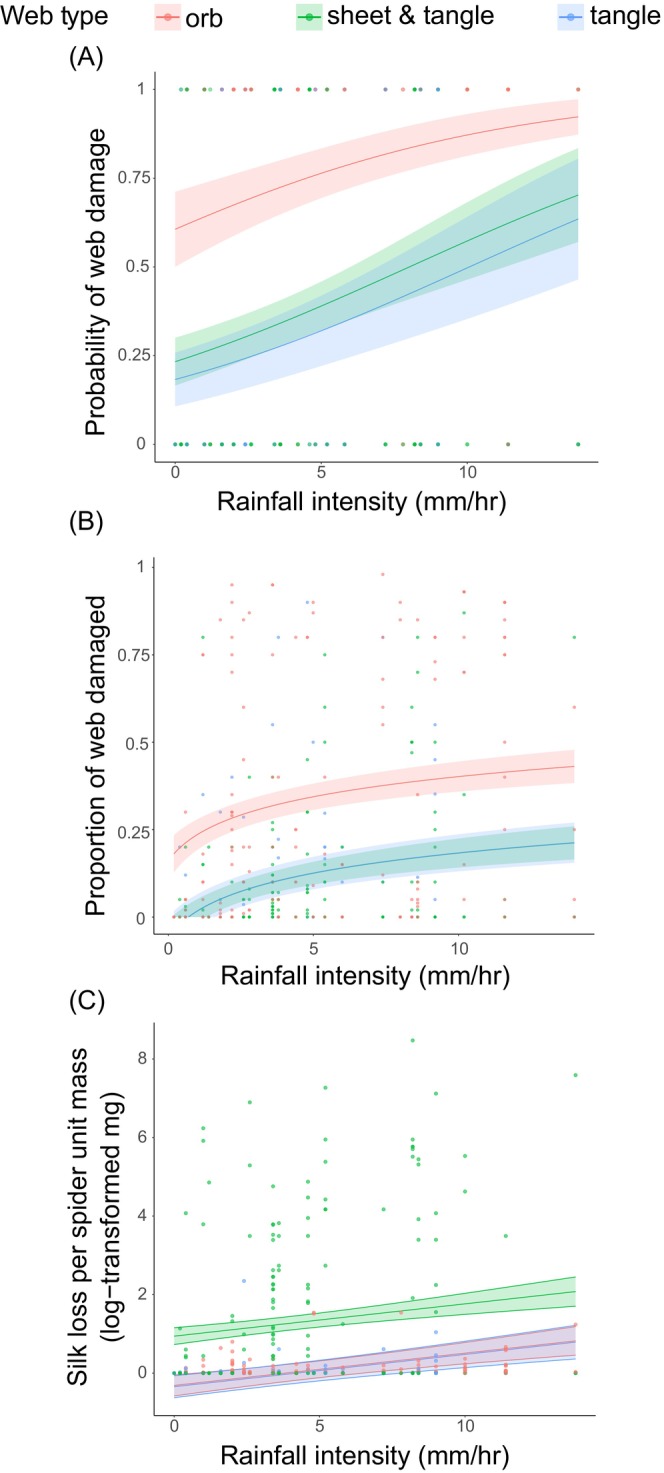
Web damage and rainfall intensity effects on different web types across a gradient of rain intensity. (A) Predicted probability of web damage as a function of rainfall intensity for each web type. (B) Model fit showing the relationship between the proportion of the web damaged and rainfall intensity for all web types. (C) Log‐transformed silk loss per unit spider mass plotted against rainfall intensity in orb webs, tangle webs, and sheet‐and‐tangle webs. The solid lines represent the fitted model, and the shaded areas indicate the 95% confidence intervals.

**FIGURE 6:**
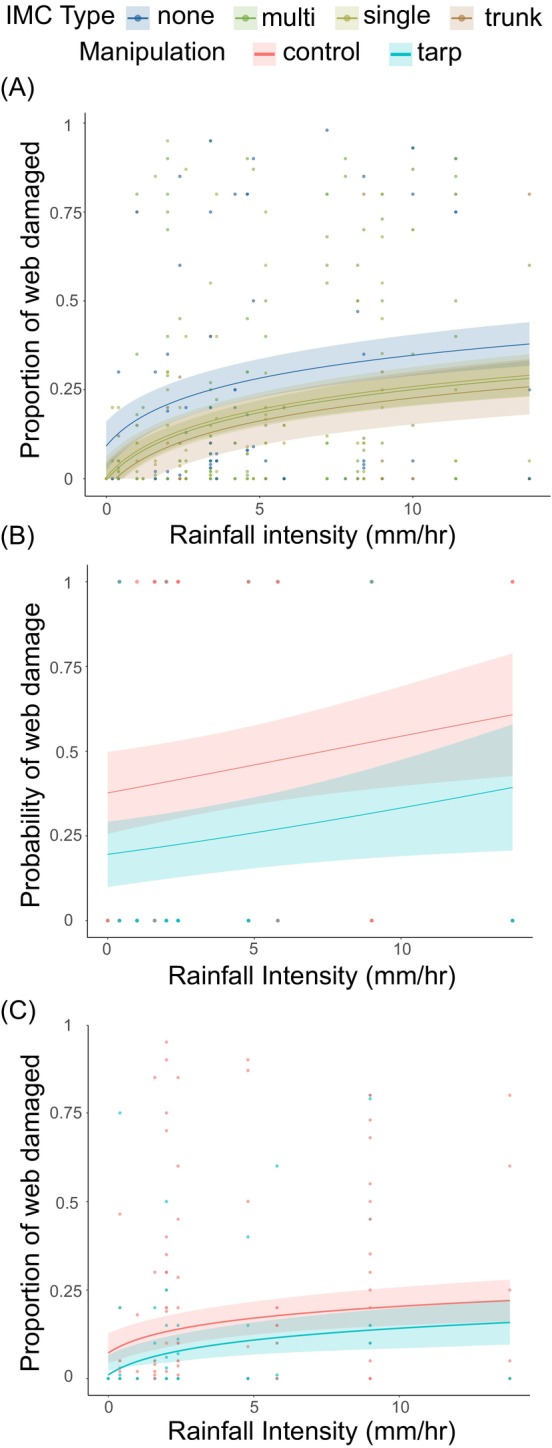
Effects of rainfall intensity (mm/h) on spider web damage as a function of immediate cover (IMC) type in the observational study and of treatment type in the manipulative experiment. (A) The solid lines represent the fitted model for different cover types (none, single leaf, multiple leaves, against trunk) with canopy cover and spider length as covariates and web ID, nested within location, as a random effect. (B) Predicted probability of web damage as a function of rainfall intensity for control and tarp‐protected webs. (C) Proportion of the web damaged as a function of rainfall intensity for control and tarp‐protected webs. The solid line represents the fitted model. The shaded areas indicate the 95% confidence intervals.

Among the three web types, orb webs had a higher probability of getting damaged by rain than the other two web types across all rain intensities (*χ* ^2^ = 38.0, *p* < 0.001, Figure 5A, Table S7). The proportion of a web that was damaged by rain increased with rainfall intensity for all web types (*χ* ^2^ = 34.4, *p* < 0.001, Figure 5B, Table S8), but was consistently greatest for orb webs (post hoc Tukey tests, Figure 5B, Table S8). However, as sheet‐and‐tangle webs consisted of considerably denser silk compared to the other two web types (Straus et al. 2022), sheet‐and‐tangle weavers suffered the greatest material loss per unit spider mass (post hoc Tukey tests, Figure 5C, Table S9). The amount of silk lost per unit mass consistently increased with rain intensity for the three web types (*χ* ^2^ = 25.8, *p* < 0.001, Figure 5C, Table S9). When assessing the rainfall damage webs suffered as a function of microhabitat type, we found that webs built in microhabitats without any immediate cover had the most damage, whereas those built against tree trunks had the least (post hoc Tukey tests, Figure 6A, Table S10).

For the manipulative experiment in the lowland tropical rainforest (Yasuní National Park, Table S1), we included 31 webs with tarp protection and 55 webs left unprotected. Unprotected webs in the control group experienced a significantly higher probability of getting damaged (*χ* ^2^ = 5.9, *p* = 0.015; Figure 6B) and showed a trend toward greater damage when compared with webs protected by tarps (*χ* ^2^ = 3.8, *p* = 0.052; Figure 6C). In both cases, the probability of the webs getting damaged and the amount of damage received increased as a function of the intensity of the rainfall events (*χ* ^2^ = 5.8, *p* = 0.016 for the probability of damage; *χ* ^2^ = 6.5, *p* = 0.011 for the proportion of the web damaged; Figures 6B,C and Tables S11 and S12, respectively).

## 4 Discussion

Abiotic factors play a major role in structuring plant and animal communities, with precipitation being key among those (Gutiérrez‐Hernández and García 2021; Srivastava et al. 2020). The rate at which rain falls (i.e., rainfall intensity), not just its total amount, is also likely to play a role in structuring plant and animal communities. Systematic studies of the impact of rain intensity on organisms and the structures they build, however, are scarce (Prein et al. 2017). Taking advantage of a gradient of precipitation intensity on the eastern slopes of the tropical Andes, we show the differential effect of rain intensity on the probability and amount of damage spider webs of different architectures suffer. After confirming a decline in rain intensity with elevation in this region of the Andes (figure 3A, see also Hoffman and Avilés 2017), we estimated the probability and amount of damage suffered by orb, tangle, and sheet‐and‐tangle webs (Table S1) and tested the hypothesis that the differences in damage among web types are partly a function of the microhabitats they occupy. Intrinsic differences in the mechanical properties and structural organization of web architectures may also influence susceptibility to rainfall damage; however, our study did not directly test mechanical resistance among web types. By being in open microhabitats (figure 4A; see also Blamires et al. 2007; Haberkern et al. 2020; Uetz et al. 1978), orb webs were more frequently damaged and lost a greater proportion of the web following rain events (Figure 5A,B). Tangle and sheet‐and‐tangle webs, which occupied more protected microhabitats, in particular the latter in wetter ecosystems (Figure 4A,C; Tables S5 and S6), were less likely to be damaged (Figure 5A,B; Tables S7 and S8). Because sheet‐and‐tangle webs contain two orders of magnitude more silk than the other two web types (Straus et al. 2022), they suffered the greatest material loss per unit spider mass of any web type (Figure 5C; Table S9). Manual rain gauges showed that rain rate (mm/h) in open microhabitats was two to three times greater than under the immediate cover of leaves or against tree trunks (Figure 3B; Table S4), consistent with the amount of damage webs suffered based on their microhabitat use (Figure 6A; Table S9). We confirmed the causal role of immediate cover in reducing damage from rainfall through a manipulative experiment where tarp‐protected webs suffered significantly less damage following rainfall events than unprotected ones (Figure 6B,C; Table S11). Altogether, our findings suggest that rain intensity, in interaction with the silk content and microhabitat use of orb, tangle and sheet‐and‐tangle webs, may play a significant role in structuring web‐building spider communities across diverse ecosystems.

We show that the challenge posed by heavy rainfall is experienced differentially by spiders with different web types. As orb weavers mostly build their webs without any immediate cover, likely to maximally intercept flying insects (Harwood et al. 2003), they are exposed to a higher probability of getting damaged by rainfall. However, by using minimal silk to construct their webs (Straus et al. 2022), repairing the damaged part (Tew et al. 2015), or ingesting silk prior to rebuilding the entire web (Blackledge 2011; Blackledge et al. 2011), these spiders minimize overall silk loss per unit of body mass (Figure 5C). In regions where heavy rainfall is common, however, 2D orb weavers may fortify their webs by increasing the toughness and strength of their silk (Hopfe et al. 2024). In contrast, 3D web‐builders appear protected by their use of microhabitats that offer greater protection from rain. Most tangle webs are built under a single or multiple leaves (Figure 4B), a pattern almost certainly resulting from their use of leaves for structural support (Harmer et al. 2011), which incidentally also provides protection from rain (Haberkern et al. 2020; Robertson and Avilés 2019). 3D sheet‐and‐tangle weavers, on the other hand, face a substantial energetic challenge because constructing their webs requires two orders of magnitude more silk per unit spider mass (Straus et al. 2022), with the quantity of silk expected to be directly proportional to the energetic cost of production (Prestwich 1977). Thus, it is even more critical for them to occupy suitable microhabitats. Compared to orb and tangle weavers, sheet‐and‐tangle weavers tended to build their webs against tree trunks (Figure 4C), especially in the lowland tropical rainforest where rainfall intensity is the greatest (e.g., Yasuní National Park and Bigal River Biological Reserve, Figure 4C and Table S2). We cannot confirm whether spiders actively choose to build against tree trunks or whether webs built against tree trunks are more likely to persist due to reduced rainfall damage. Nonetheless, building webs against tree trunks could provide further benefits, for instance, through structural support for a larger web (Harmer et al. 2011) and offering greater canopy cover (Figure 4E) for additional protection from rainfall.

The capacity of web weavers to minimize or offset the high energetic costs of silk production may contribute to their persistence and resilience in environments with high rainfall intensity. Because silk production represents a substantial metabolic investment, the relationship between body size and mass‐specific metabolic rate is likely to influence the relative cost of web construction and repair. Tangle weavers, for instance, tend to have small body sizes (Craig 1987; Greenberg‐Pines et al. 2024). Smaller organisms generally exhibit higher mass‐specific metabolic rates than larger ones (Brown et al. 2004; Makarieva et al. 2008), potentially increasing the relative energetic burden of silk production. The small webs tangle weavers build, however, can more easily gain protection from the rain under the immediate cover of leaves (Figure 4D). Such protection may reduce rainfall‐induced damage and thus lower the energetic costs associated with frequent web repair or reconstruction. A reduction in relative energy consumption per unit body mass with increasing size, referred to as hypometric scaling and demonstrated across a wide range of organisms, from plants to animals (Brown et al. 2004; Enquist 2002; Gillooly et al. 2010; Glazier 2005; Makarieva et al. 2008), should lower per‐unit‐mass energetic costs of web construction and repair in larger spiders (Greenberg‐Pines et al. 2024). In lowland tropical rainforests, where the likelihood of rainfall‐induced web damage is highest, 3D sheet‐and‐tangle weavers tend to be relatively large spiders, such as species in the genus *Aglaoctenus* (Family Lycosidae) with body lengths ranging from 1.3 to 1.5 cm, the largest recorded in our study. The other main sheet‐and‐tangle builders in the lowland tropical rainforests are group‐living spiders in the genus *Anelosimus* (Family Theridiidae). Group living has been suggested as an alternative strategy to address the need for constant web repair in habitats where webs are frequently damaged by strong rains (Avilés and Guevara 2017; Hoffman and Avilés 2017; Purcell and Avilés 2008). With multiple individuals sharing the costs of web maintenance and repair, group‐living spiders have been shown to reduce per capita costs via economies of scale (Straus et al. 2022), making it possible for them to afford the higher energetic costs associated with web construction and repair in environments with intense rainfall.

As mentioned in the Introduction, rainfall can affect ecosystems in both positive and negative ways. Because plants need water for development, photosynthesis, and survival, increased precipitation enhances regional primary productivity (Hsu et al. 2012; Knapp et al. 2001). Higher plant productivity, in turn, has been shown to monotonically increase species richness across trophic levels (Wimp et al. 2010). Such an increase is likely associated with greater insect abundance, which in turn would increase prey availability for predator guilds, such as spiders. Lusher vegetation may also offer improved structural support as well as increased rain protection given that average leaf area increases with precipitation (Robertson and Avilés 2019; Törnros and Menzel 2014).

On the other hand, heavy rainfall can negatively affect organismal fitness by damaging the structures organisms build (i.e., their extended phenotypes), as well as by challenging their survival and foraging opportunities. Bird nests, for instance, are extended phenotypes built to provide safe and thermally stable environments for eggs and chicks to develop. Heavy and persistent rainfall, however, can lead to nest failure and reduce overall brood survival (Anctil et al. 2014; Schöll and Hille 2020). Other extended phenotypes that may be affected by extreme rain include the nests social wasps built. Wasps in the genus *Polybia*, for instance, select nesting sites that reduce impact from adverse weather (Dejean et al. 2010); nonetheless, heavy rainfall during La Niña years has been shown to have a detrimental effect on wasp populations (Dejean et al. 2011). In terms of direct effects, small insects, such as mosquitoes, may have higher mortality when stranding in water and drowning (Dickerson et al. 2012). Larger insects, such as butterflies or honeybees, may also be affected by being more likely to be splashed and experiencing greater impact force from raindrops than smaller insects, thus also requiring shelter from precipitation (Dickerson et al. 2014; He et al. 2016; Pardikes et al. 2015).

The direct or indirect effects of rainfall on organisms and their extended phenotypes may, in turn, have cascading effects across trophic levels. By reducing insect activity, for instance, rainfall events may negatively affect aerial insectivores, such as birds (Cox et al. 2019), bats (Davy et al. 2022), and spiders. By assessing prey community composition, availability, and rates of prey capture before, during, and after rainfall events, web‐building spiders may serve as model systems to explore such cascading effects. Such assessments would further help understand the relationships among energy supply, web construction costs, and microhabitat selection, as well as help explain how sheet‐and‐tangle weavers sustain the exceptionally high energetic costs of web construction. Expanding this work to other taxa would further elucidate the multifaceted impacts of rainfall on multi‐trophic interactions, including plant productivity, herbivory, predation, facilitation, and competition or mutualism, all of which ultimately shape community dynamics (França et al. 2020; McCluney et al. 2012).

In summary, we argue that web‐building spiders with their diverse web architectures provide an ideal system for investigating how abiotic factors influence organismal fitness and, thus, community structure. Our observational study revealed the intricate interplay among rainfall intensity, web architecture, and microhabitat use in web‐building spiders, with our manipulative experiment providing support for the hypothesis that immediate cover can effectively moderate the effects of strong rainfall (Robertson and Avilés 2019; Haberkern et al. 2020). Our findings further point to how differences in silk content among different web types may influence the taxonomic and functional composition of web‐building spider communities across precipitation gradients. More generally, by underscoring the importance of the interaction between abiotic stressors and the structure and function of organisms, our studies provide insights for predicting changes in ecological communities under changing climatic conditions.

## Author Contributions

**Yu‐Heng Lin:** conceptualization (lead), formal analysis (lead), investigation (equal), methodology (lead), writing – original draft (lead), writing – review and editing (equal). **Antonio Domingos Brescovit:** data curation (lead), writing – review and editing (equal). **Leticia Avilés:** conceptualization (lead), formal analysis (equal), funding acquisition (lead), methodology (equal), validation (equal), writing – original draft (lead), writing – review and editing (equal).

## Funding

This work was supported by the Natural Sciences and Engineering Research Council of Canada DGs RGPIN‐2019‐06539 and RGPIN‐2025‐06896, the Centros de Pesquisa, Inovação e Difusão, Fundação Amazônia Paraense de Amparo à Pesquisa, 2022/12588‐1, the Conselho Nacional de Desenvolvimento Cientifico e Tecnologico, 3003903/2019‐8, and a student research grant from the American Arachnological Society.

## Conflicts of Interest

The authors declare no conflicts of interest.

## Supporting information


**Figure S1:** Maximum‐likelihood phylogeny of spider families that contain web building genera. The phylogeny was reconstructed using ultraconserved elements (UCEs) with a 25% occupancy threshold, as reported by Kulkarni et al. (2023). We used the IQ‐Tree algorithm (Nguyen et al., 2015) to perform the phylogenomic analyses following the model (GTR + I + F + G4), as selected in Kulkarni et al. (2023). We then pruned the phylogeny to include only the families represented in our dataset. This tree was used to define phylogenetically separate groups of taxa that could serve as replicates for the statistical analyses. Colored branches indicate three phylogenetically separate groups (non‐overlapping branches; Felsenstein, 1985; Maddison, 2000), each containing orb, tangle, and sheet‐and‐tangle web types.
**Table S1:** Family and genera of spiders with two‐dimensional (2D) or three dimensional (3D) webs included in this study. Spiders were grouped based on the architecture of their webs, which not always corresponded with what's expected from their taxonomy. Thus, *Kapogea*, which belongs to a predominantly orb‐weaving family, builds a sheet‐and‐tangle web structure, whereas Architis, Dossenus, and Aglaoctenus are web‐building genera within typically wandering spider families. Listed are also number of specimens, their elevational range, immediate cover type (IMC: no cover (N), multiple‐leaf (M), single‐leaf (S), against trunk(T)), and range of responses to web damage (move = abandon site following web damage; rebuild = rebuild web at original site after removing web remains; repair = repair damaged portion of the web).
**Table S2:** Characteristics of the sites along the elevational gradient in eastern Ecuador where the effects of rain intensity on spider webs were investigated.
**Table S3:** Statistical models used in each table and figure.
**Table S4:** Rainfall intensity under different immediate cover types along the eastern slopes of the tropical Andes. Values were estimated by dividing the total amount of rain collected in the manual rain gauges during a given day by the duration of the active rainfall events during that day at their respective site. The linear regression model (glmmTMB package), followed by Tukey tests, included elevation, which correlates with rain intensity (Figure 3A), as a co‐variate. Boldface denotes significant differences; letter codes show results of Tukey tests.
**Table S5:** Test of the differences among web types on the percentage of a web's horizontal projection covered by some type of immediate cover (within 25 cm above the web) across the elevational gradient (glmmTMB package). Boldface denotes significant differences in a beta regression model with elevation as a covariate and three phylogenetically separate groups of taxa as a random factor; letter codes show results of Tukey tests.
**Table S6:** Test of differences among spider web types on the amount of canopy cover above the webs (percent cover) across the elevational gradient (glmmTMB package). Boldface denotes significant predictors in a beta regression model with elevation as a covariate and three phylogenetically separate groups of taxa as a random factor; letter codes show results of Tukey tests.
**Table S7:** Probability of webs being damaged as a function of web type and rain intensity (mm/h) along an elevational gradient on the eastern tropical Andes. Results of a logistic regression (glmmTMB, binomial) with spider length as a covariate and random effects for web ID, nested within site, and three phylogenetically separate groups of taxa. Boldface denotes significant predictors; letter codes show results of Tukey tests.
**Table S8:** Tests of the effect of rain intensity (mm/h) on the percent web damage suffered by spiders of different web types in the observational study. Results of a GLMM with a beta‐transformed distribution, including spider length as a covariate and random effects for web ID, nested within site, and three phylogenetically separate groups of taxa (glmmTMB package). Boldface denotes significant predictors; letter codes show results of Tukey tests.
**Table S9:** Tests of web silk loss per unit spider mass (mg) as a function of web type and rain intensity along an elevational gradient in the observational study (see Methods in main paper for silk loss per unit spider mass calculations). Log‐transformed silk loss per unit spider mass as a function of rainfall intensity across web types in a GLMM with spider length as a covariate and random effects for web ID, nested within site, and three phylogenetically separate groups of taxa (glmmTMB package). Boldface denotes significant predictors; letter codes show results of Tukey tests.
**Table S10:** Effect of rain intensity (mm/h) on the amount of damage webs suffered as a function of immediate cover (IMC) type along a precipitation gradient on the tropical Andes. Results of GLMM with a beta‐transformed distribution, including canopy cover and spider length as a covariate and random effects for web ID, nested within site, and three phylogenetically separate groups of taxa (glmmTMB package). Boldface denotes significant predictors; letter codes show results of Tukey tests.
**Table S11:** Results of a manipulative experiment to test the effect of immediate cover on the probability of rainfall damage as a function of web type and rainfall intensity at a lowland tropical rainforest site on the eastern slopes of the tropical Andes (Yasuní, 247 m elevation and 3.31 mm/h average rain intensity). Probability of web damage in a logistic regression with spider length as a covariate and random effects for web ID and three phylogenetically separate groups of taxa (glmmTMB package). Boldface denotes significant predictors; letter codes show results of Tukey tests.
**Table S12:** Results of a manipulative experiment to test the effect of immediate cover on the amount of web damage as a function of web type and rain intensity at a lowland tropical rainforest site on the eastern slopes of the tropical Andes (Yasuní, 247 m elevation and 3.31 mm/h rain intensity). Log‐transformed web damage as a function of treatment type, rainfall intensity, and web type in a GLMM with spider length as a covariate and random effects for web ID and three phylogenetically separate groups (glmmTMB package). Boldface denotes significant predictors; letter codes show results of Tukey tests.

## Data Availability

All code and data are available and archived on Zenodo (https://doi.org/10.5281/zenodo.18026100).
