# Supplementary material for: Spider Web Architecture and Rainfall Damage: Observational and Manipulative Studies Along a Precipitation Gradient on the Tropical Andes
Source: Ecol Evol. 2026 Apr 7;16(4):e73432. doi: 10.1002/ece3.73432 (PMC13054244; doi:10.1002/ece3.73432)
Supplement: Supplementary file 1 — Figure S1: Maximum‐likelihood phylogeny of spider families that contain web building genera. The phylogeny was reconstructed using ultraconserved elements (UCEs) with a 25% occupancy threshold, as reported by Kulkarni et al. (2023). We used the IQ‐Tree algorithm (Nguyen et al., 2015) to perform the phylogenomic analyses following the model (GTR + I + F + G4), as selected in Kulkarni et al. (2023). We then pruned the phylogeny to include only the families represented in our dataset. This tree was used to define phylogenetically separate groups of taxa that could serve as replicates for the statistical analyses. Colored branches indicate three phylogenetically separate groups (non‐overlapping branches; Felsenstein, 1985; Maddison, 2000), each containing orb, tangle, and sheet‐and‐tangle web types. Table S1: Family and genera of spiders with two‐dimensional (2D) or three dimensional (3D) webs included in this study. Spiders were grouped based on the architecture of their webs, which not always corresponded with what's expected from their taxonomy. Thus, Kapogea, which belongs to a predominantly orb‐weaving family, builds a sheet‐and‐tangle web structure, whereas Architis, Dossenus, and Aglaoctenus are web‐building genera within typically wandering spider families. Listed are also number of specimens, their elevational range, immediate cover type (IMC: no cover (N), multiple‐leaf (M), single‐leaf (S), against trunk(T)), and range of responses to web damage (move = abandon site following web damage; rebuild = rebuild web at original site after removing web remains; repair = repair damaged portion of the web). Table S2: Characteristics of the sites along the elevational gradient in eastern Ecuador where the effects of rain intensity on spider webs were investigated. Table S3: Statistical models used in each table and figure. Table S4: Rainfall intensity under different immediate cover types along the eastern slopes of the tropical Andes. Values were estimated by dividing [file ECE3-16-e73432-s001.pdf]

SUPPORTING INFORMATION for:

Spider web architecture and rainfall damage: observational and manipulative studies along a precipitation gradient on the tropical Andes

Ecology and Evolution, 2026

Authors: Yu-Heng Lin[1*,] Antonio Domingos Brescovit[2], Leticia Avilés[1*]

Affiliations and contact information:

[1]Department of Zoology and Biodiversity Research Centre, University of British Columbia, Vancouver, BC, Canada

[2]Laboratório de Coleções Zoológicas, Instituto Butantan, São Paulo, SP, Brazil

*Corresponding author: Yu-Heng Lin, email: yh.lin@zoology.ubc.ca

Leticia Avilés, email: laviles@zoology.ubc.ca

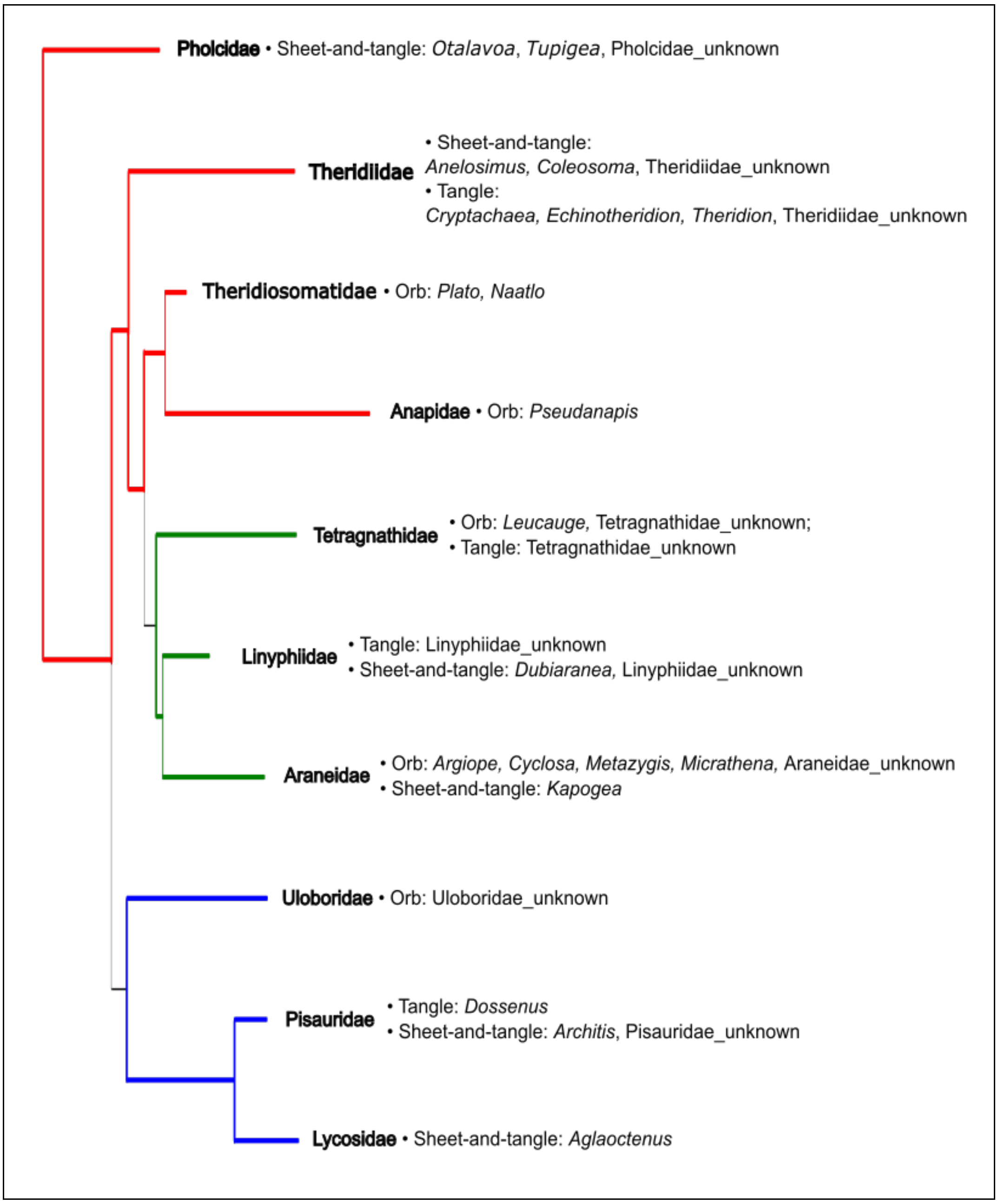

Figure S1. Maximum-likelihood phylogeny of spider families that contain web-building genera. The phylogeny was reconstructed using ultraconserved elements (UCEs) with a 25% occupancy threshold, as reported by Kulkarni et al. (2023). We used the *IQ-Tree* algorithm (Nguyen et al., 2015) to perform the phylogenomic analyses following the model (GTR + I + F + G4), as selected in Kulkarni et al., 2023. We then pruned the phylogeny to include only the families represented in our dataset. This tree was used to define phylogenetically separate groups of taxa that could serve as replicates for the statistical analyses. Coloured branches indicate three phylogenetically separate groups (non-overlapping branches; Felsenstein, 1985; Maddison, 2000), each containing orb, tangle, and sheet-and-tangle web types.

Table S1. Family and genera of spiders with two-dimensional (2D) or three-dimensional (3D) webs included in this study. Spiders were grouped based on the architecture of their webs, which not always corresponded with what's expected from their taxonomy. Thus, *Kapogea*, which belongs to a predominantly orb-weaving family, builds a sheet-and-tangle web structure, whereas *Architis*, *Dossenus*, and *Aglaoctenus* are web-building genera within typically wandering spider families. Listed are also number of specimens, their elevational range, immediate cover type (IMC: no cover (N), multiple-leaf (M), single-leaf (S), against trunk(T)), and range of responses to web damage (move = abandon site following web damage; rebuild = rebuild web at original site after removing web remains; repair = repair damaged portion of the web).

Web type	Family	Genus	# specimens	Elevational range	IMC	Damage responses
Orb	Anapidae	*Pseudanapis*	1	958	N	Repair
	Araneidae	*Argiope*	2	247	M, S	Rebuild
		Cyclosa	12	247-2170	M, S	Move, repair
		Metazygia	1	247	M	Repair
		Micrathena	3	247-2170	N	Move, rebuild, repair
		Unknown	3	247-2170	N, M, S	Repair
	Tetragnathidae	*Leucauge*	10	958-2170	N, M	Move, rebuild, repair
		Unknown	3	2170-3439	N, M, S	Remove, repair
	Theridiosomatidae	*Plato*	1	958	S	Rebuild
		Naatlo	2	958	N	Repair
	Uloboridae	Unknown	3	247-1178	N, M	Repair
Tangle	Linyphiidae	Unknown	2	3439	M, S	Repair
	Pisauridae	*Dossenus*	1	247	M	Repair
	Tetragnathidae	Unknown	1	1178	M	Repair
	Theridiidae	*Cryptachaea*	2	1178	M, S	Repair
		Echinotheridion	1	247	S	Repair

		Theridion	1	247	S	Repair
		Unknown	10	247-2170	M, S, T	Repair
Sheet-and-tangle	Araneidae	*Kapogea*	2	247	M, T	Repair
	Linyphiidae	*Dubiaranea*	8	2170-3439	N, M	Repair
		Unknown	7	2170-3439	M, S	Move, repair
	Lycosidae	*Aglaoctenus*	7	247	M, T	Repair
	Pholcidae	*Otalavoa*	1	1178	S	Repair
		Tupigea	2	958-1178	N, M	Repair
		Unknown	20	247-1178	N, M, S, T	Repair
	Pisauridae	*Architis*	3	247	N, S, T	Rebuild, repair
		Unknown	2	958	M	Repair
	Theridiidae	*Anelosimus*	2	247	M	Repair
		Coleosoma	1	247	M	Repair
		Unknown	7	1178-3439	M, S, T	Repair

Table S2. Characteristics of the sites along the elevational gradient in eastern Ecuador where the effects of rain intensity on spider webs were investigated.

Study site	Elevation (m)	Latitude	Longitude	Rainfall intensity (mm/hr)	Dates (2022)
Yasuní	247	S0.6741	W76.3970	3.31	June 4th – July 2nd
Bigal	958	S0.256	W77.1958	4.13	July 18th – July 23rd
Narupa	1178	S0.6935	W77.7261	2.12	July 12th – July 16th
Yanayacu	2170	S0.4619	W77.4527	2.87	July 6th – July 11th
Cayambe-Coca	3439	S0.2931	W78.0636	1.27	July 25th – July 28th

Table S3. Statistical models used in each table and figure.

Figure	Table	Model
Figure 3A	NA	lm(rain_rate ~ scale(Elevation))
Figure 3B	Table S4	lm(rain_rate ~ scale(Elevation) + IMC)
Figure 4D	Table S5	glmmTMB(IMC% ~ Web_Type + scale(Elevation) + (1|Phylogenetically_separate_group), family = beta_family)
Figure 4E	Table S6	glmmTMB(CanopyCover% ~ Web_Type + scale(Elevation) + (1|Phylogenetically_separate_group), family = beta_family)
Figure 5A	Table S7	glmmTMB(Dmg_yes/no ~ scale(rain intensity) + Web_Type + scale(Spider_Length) + (1 | Location/Web ID) + (1|Phylogenetically_separate_group), family = binomial())
Figure 5B	Table S8	glmmTMB(Dmg% ~ log_rain intensity + Web_Type + scale(Spider_Length) + (1 | Location/ Web ID) + (1|Phylogenetically_separate_group), family = beta_family)
Figure 5C	Table S9	glmmTMB(log_dmg_per_unit_mass ~ scale(rain intensity) + Web_Type + scale(Spider_Length) + (1 | Location/ Web ID)) + (1|Phylogenetically_separate_group))
Figure 6A	Table S10	glmmTMB(Dmg% ~ log_rain intensity + IMC_Type + CanopyCover% + scale(Spider_Length) + (1 | Location/ Web ID) + (1|Phylogenetically_separate_group), family = beta_family)
Figure 6B	Table S11	glmmTMB(Dmg_yes/no~ Manipulation + Web_Type + scale(rain intensity) + scale(Spider_Length) + (1| Web_ID) + (1|Phylogenetically_separate_group), family = binomial())
Figure 6C	Table S12	glmmTMB(Dmg%~Manipulation + Web_Type + log_rain intensity + scale(Spider_Length) + (1| Web_ID) + (1|Phylogenetically_separate_group), family = beta_family)

Table S4. Rainfall intensity under different immediate cover types along the eastern slopes of the tropical Andes. Values were estimated by dividing the total amount of rain collected in the manual rain gauges during a given day by the duration of the active rainfall events during that day at their respective site. The linear regression model (glmmTMB package), followed by Tukey tests, included elevation, which correlates with rain intensity (Fig. 3A), as a co-variate. **Boldface** denotes significant differences; letter codes show results of Tukey tests.

Full model	Estimate	SE	t value	P	Post hoc Tukey test
**(Intercept)**	**0.80**	**0.206**	**3.90**	**<0.001**	
Elevation	-0.17	0.151	-1.10	0.27	
(No cover as a standard for the comparison)					A
**Multi**	**-0.77**	**0.182**	**-4.26**	**<0.001**	B
**Single**	**-1.26**	**0.194**	**-6.47**	**<0.001**	B
**Trunk**	**-0.77**	**0.181**	**-4.27**	**<0.001**	B

Table S5. Test of the differences among web types on the percentage of a web's horizontal projection covered by some type of immediate cover (within 25 cm above the web) across the elevational gradient (glmmTMB package). **Boldface** denotes significant differences in a beta regression model with elevation as a covariate and three phylogenetically separate groups of taxa as a random factor; letter codes show results of Tukey tests.

Full model	Estimate	SE	Z value	P	Post hoc Tukey test
**(Intercept)**	**-1.18**	**0.138**	**-8.55**	**<0.001**	
Elevation	-0.06	0.081	-0.77	0.441	
(Orb webs as a standard for the comparison)					A
Sheet-and-tangle	0.34	0.178	1.90	0.057	A
**Tangle**	**1.18**	**0.251**	**4.71**	**<0.001**	B

Table S6. Test of differences among spider web types on the amount of canopy cover above the webs (percent cover) across the elevational gradient (glmmTMB package). **Boldface** denotes significant predictors in a beta regression model with elevation as a covariate and three phylogenetically separate groups of taxa as a random factor; letter codes show results of Tukey tests.

Full model	Estimate	SE	Z value	P	Post hoc Tukey test
**(Intercept)**	**0.66**	**0.085**	**7.763**	**<0.001**	
**Elevation**	**-0.14**	**0.056**	**-2.494**	**0.013**	
(Orb webs as a standard for the comparison)					A
**Sheet-and-tangle**	**0.30**	**0.12**	**2.514**	**0.012**	B
Tangle	-0.01	0.159	-0.044	0.965	AB

Table S7. Probability of webs being damaged as a function of web type and rain intensity (mm/hr) along an elevational gradient on the eastern tropical Andes**.** Results of a logistic regression (glmmTMB, binomial) with spider length as a covariate and random effects for web ID, nested within site, and three phylogenetically separate groups of taxa. **Boldface** denotes significant predictors; letter codes show results of Tukey tests.

Full model	Estimate	SE	Z value	P	Post hoc Tukey test
**(Intercept)**	**1.46**	**0.605**	**2.42**	**0.016**	
**Rainfall intensity**	**0.73**	**0.136**	**5.34**	**<0.001**	
Spider length	0.38	0.203	1.86	0.062	
(Orb webs as a standard for the comparison)					A
**Sheet-and-tangle**	**-2.32**	**0.412**	**-5.64**	**<0.001**	B
**Tangle**	**-2.61**	**0.494**	**-5.28**	**<0.001**	B

Table S8. Tests of the effect of rain intensity (mm/hr) on the percent web damage suffered by spiders of different web types in the observational study. Results of a GLMM with a beta-transformed distribution, including spider length as a covariate and random effects for web ID, nested within site, and three phylogenetically separate groups of taxa (glmmTMB package). **Boldface** denotes significant predictors; letter codes show results of Tukey tests.

Full model	Estimate	SE	Z value	P	Post hoc Tukey test
**(Intercept)**	**-1.66**	**0.193**	**-8.57**	**<0.001**	
**Rainfall intensity**	**0.38**	**0.064**	**5.86**	**<0.001**	
Spider length	0.05	0.069	0.69	0.489	
(Orb webs as a standard for the comparison)					A
**Sheet-and-tangle**	**-0.87**	**0.132**	**-6.61**	**<0.001**	B
**Tangle**	**-0.97**	**0.171**	**-5.66**	**<0.001**	B

Table S9. Tests of web silk loss per unit spider mass (mg) as a function of web type and rain intensity along an elevational gradient in the observational study (see Methods in main paper for silk loss per unit spider mass calculations). Log-transformed silk loss per unit spider mass as a function of rainfall intensity across web types in a GLMM with spider length as a covariate and random effects for web ID, nested within site, and three phylogenetically separate groups of taxa (glmmTMB package). **Boldface** denotes significant predictors; letter codes show results of Tukey tests.

Full model	Estimate	SE	Z value	P	Post hoc Tukey test
**(Intercept)**	0.07	0.21	0.33	0.741	
**Rainfall intensity**	**0.30**	**0.06**	**5.08**	**<0.001**	
**Spider length**	**-0.34**	**0.09**	**-3.81**	**0.001**	
(Orb webs as a standard for the comparison)					A
**Sheet-and-tangle**	**1.23**	**0.175**	**7.02**	**<0.001**	B
Tangle	-0.33	0.228	-1.45	0.147	A

Table S10. Effect of rain intensity (mm/hr) on the amount of damage webs suffered as a function of immediate cover (IMC) type along a precipitation gradient on the tropical Andes. Results of GLMM with a beta-transformed distribution, including canopy cover and spider length as a covariate and random effects for web ID, nested within site, and three phylogenetically separate groups of taxa (glmmTMB package). **Boldface** denotes significant predictors; letter codes show results of Tukey tests.

Full model	Estimate	SE	Z value	P	Post hoc Tukey test
**(Intercept)**	**-2.00**	**0.269**	**-7.43**	**<0.001**	
**Rainfall intensity**	**0.36**	**0.066**	**5.38**	**<0.001**	
Canopy cover percentage	-0.00	0.003	-1.66	0.098	
Spider length	0.05	0.063	0.84	0.401	
(No cover as a standard for the comparison)					A
**Multi**	**-0.52**	**0.152**	**-3.38**	**0.004**	B
Single	-0.37	0.17	-2.18	0.129	AB
**Trunk**	**-0.65**	**0.206**	**-3.16**	**0.009**	B

Table S11. Results of a manipulative experiment to test the effect of immediate cover on the probability of rainfall damage as a function of web type and rainfall intensity at a lowland tropical rainforest site on the eastern slopes of the tropical Andes (Yasuní, 247 m elevation and 3.31 mm/hr average rain intensity). Probability of web damage in a logistic regression with spider length as a covariate and random effects for web ID and three phylogenetically separate groups of taxa (glmmTMB package). **Boldface** denotes significant predictors; letter codes show results of Tukey tests.

Full model	Estimate	SE	Z value	P	Post hoc Tukey test
**(Intercept)**	**1.54**	**0.577**	**2.66**	**0.008**	
**Manipulation**	**-1.24**	**0.512**	**-2.42**	**0.015**	
**Rainfall intensity**	**0.41**	**0.168**	**2.42**	**0.016**	
Spider length	0.11	0.367	0.29	0.769	
(Orb webs as a standard for the comparison)					A
**Sheet-and-tangle**	**-2.14**	**0.732**	**-2.93**	**0.003**	B
**Tangle**	**-3.52**	**0.823**	**-4.27**	**<0.001**	B

Table S12. Results of a manipulative experiment to test the effect of immediate cover on the amount of web damage as a function of web type and rain intensity at a lowland tropical rainforest site on the eastern slopes of the tropical Andes (Yasuní, 247 m elevation and 3.31 mm/hr rain intensity). Log-transformed web damage as a function of treatment type, rainfall intensity, and web type in a GLMM with spider length as a covariate and random effects for web ID and three phylogenetically separate groups (glmmTMB package). **Boldface** denotes significant predictors; letter codes show results of Tukey tests.

Full model	Estimate	SE	Z value	P	Post hoc Tukey test
**(Intercept)**	**-1.73**	**0.208**	**-8.33**	**<0.001**	
Manipulation	-0.27	0.138	-1.94	0.052	
**Rainfall intensity**	**0.20**	**0.077**	**2.56**	**0.011**	
Spider length	-0.01	0.099	-0.12	0.902	
(Orb webs as a standard for the comparison)					A
**Sheet-and-tangle**	**-0.70**	**0.231**	**-3.05**	**0.002**	B
**Tangle**	**-0.93**	**0.219**	**-4.27**	**<0.001**	B

References

1. Felsenstein, J. (1985). Phylogenies and the comparative method. *The American Naturalist*, *125*(1), 1–15.
2. Kulkarni, S., Wood, H. M., & Hormiga, G. (2023). Advances in the reconstruction of the spider tree of life: A roadmap for spider systematics and comparative studies. *Cladistics*, *39*(6), 479–532.
3. Maddison, W. P. (2000). Testing character correlation using pairwise comparisons on a phylogeny. *Journal of Theoretical Biology*, *202*(3), 195–204.
4. Nguyen, L.-T., Schmidt, H. A., Von Haeseler, A., & Minh, B. Q. (2015). IQ-TREE: a fast and effective stochastic algorithm for estimating maximum-likelihood phylogenies. *Molecular Biology and Evolution*, *32*(1), 268–274.
